# Supplementary material for: The epigenetic regulator Mll1 is required for Wnt-driven intestinal tumorigenesis and cancer stemness
Source: Nat Commun. 2020 Dec 21;11:6422. doi: 10.1038/s41467-020-20222-z (PMC7752919; doi:10.1038/s41467-020-20222-z)
Supplement: Supplementary file 1 — Supplementary Information [file 41467_2020_20222_MOESM1_ESM.pdf]

Supplementary Information

Grinat, Heuberger et al.

The epigenetic regulator Mll1 is required for Wnt-driven intestinal tumorigenesis and cancer stemness

Supplementary Figures

Supplementary Fig. 1: High Mll1 expression in colon carcinomas and intestinal crypts.

Supplementary Fig. 2: β-catenin^{GOF}-induced intestinal tumorigenesis depends on Mll1.

Supplementary Fig. 3: Lineage tracing of Mll1-deficient Lgr5^{+} intestinal stem cells.

Supplementary Fig. 4: Mll1 sustains stemness and prevents differentiation of colon cancer cells.

Supplementary Fig. 5: Mll1 regulates specific stem cell genes.

Supplementary Tables

Supplementary Table 1: Primer sequences for ChIP-qPCR.

Supplementary Table 2: Primer sequences for qRT-PCR.

Supplementary Table 3: Human CRC transcriptomic datasets.

Supplementary References

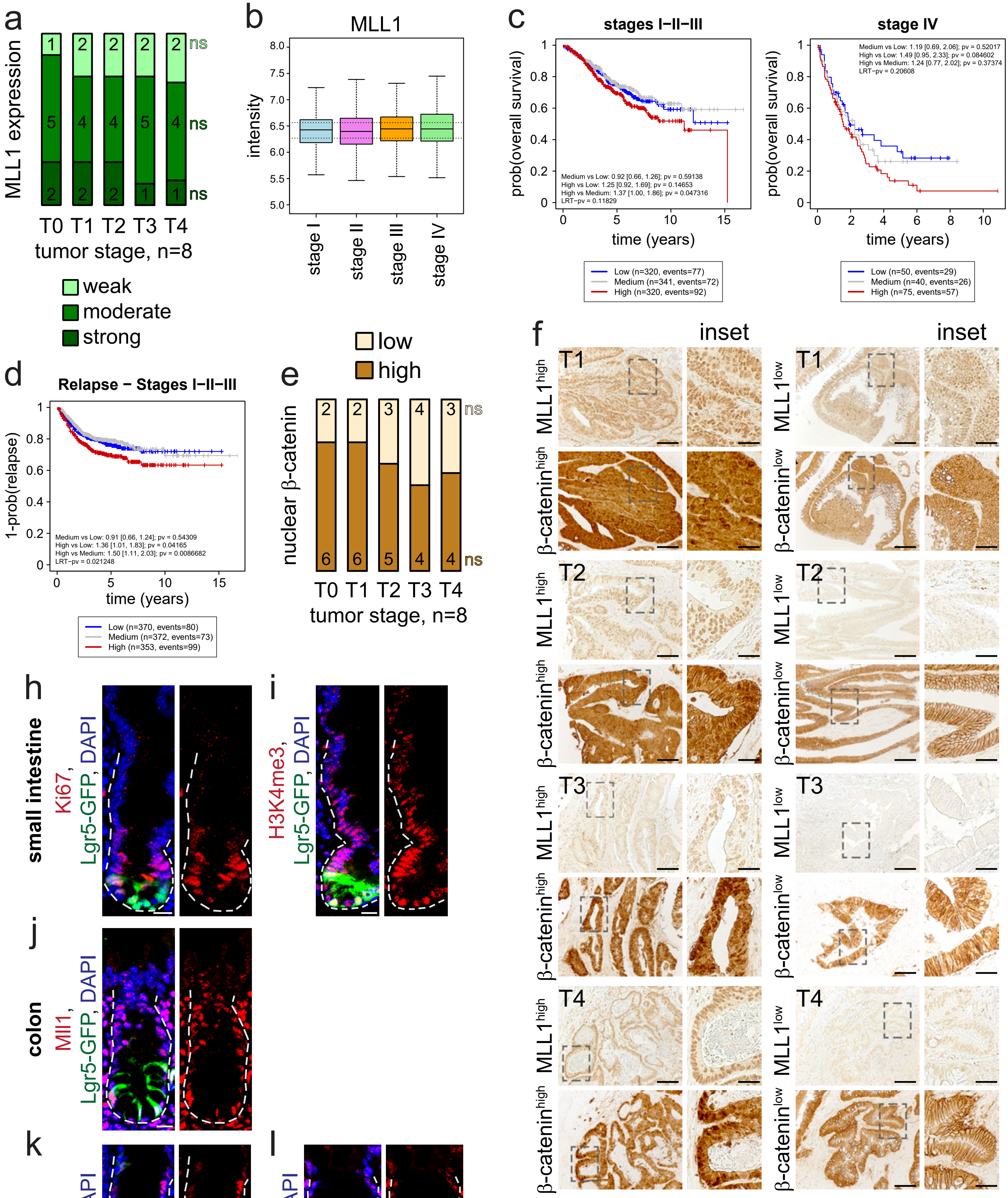

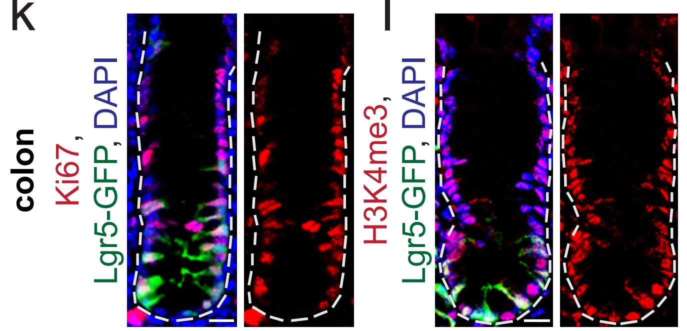

g

nuclear β-catenin	nuclear MLL1			
	weak	mod.	strong	total (n)
low	7	7	0	14
high	2	15	8	25
total (n)	9	22	8	39

Supplementary Fig. 1. **High Mll1 expression in colon carcinomas and intestinal crypts.**

a) Quantification of MLL1 stainings scored as weak, moderate and strong across human colon carcinomas: tumor stages T0-T4, n=8 primary tumors per stage. Linear regression for trend analysis. **b)** Expression analysis of *MLL1* in CRC patient tumors of AJCC stages I-IV, n=2,207 primary tumor samples. Box plots indicate median (middle line), 25th, 75th percentile (box) and 5th and 95th percentile (whiskers) as well as outliers. **c)** Kaplan-Meier plot representation of overall survival over time for patients bearing colon cancers at stages I-III (pre-metastatic, right, n=981 tumors) and IV (metastatic, left, n=165 tumors) with high, medium or low expression of *MLL1*. **d)** Kaplan-Meier plot representation of relapse risk over time for patients bearing colon cancers at stages I-III with high, medium or low expression of *MLL1*, n=1,095 samples. **e)** Quantification of β-catenin stainings scored as high and low across human colon carcinomas: tumor stages T0-T4, n=8 primary tumors per stage. Linear regression for trend analysis. **f)** Representative immunohistochemistry stainings for MLL1 (upper panels) and β-catenin (lower panels) of colon cancer patient biopsies: tumor stages T1-T4, scale bars 50μm. Insets on the right, scale bars 20μm. **g)** Associations between nuclear β-catenin and MLL1 expression in human colon carcinomas across tumor stages T0-T4, determined by immunohistochemistry analysis, n=39. **h)** Immunofluorescence for Ki67 (red) and Lgr5-GFP (green) on sections of the small intestine of Lgr5-GFP mice. **i)** H3K4me3 (red) and Lgr5-GFP (green) staining on the small intestine of Lgr5-GFP mice. **j)** Immunofluorescence for Mll1 (red) and Lgr5-GFP (green) in colon crypts of Lgr5-GFP mice. **k)** Ki67 (red) and Lgr5-GFP (green) staining for proliferative cells in the colon. **l)** H3K4me3 (red) and Lgr5-GFP (green) staining on the colon of Lgr5-GFP mice. Nuclei stained in blue (DAPI), scale bars 20μm. Immunofluorescence stainings were performed in at least three independent mice. Source data are provided as a Source Data file.

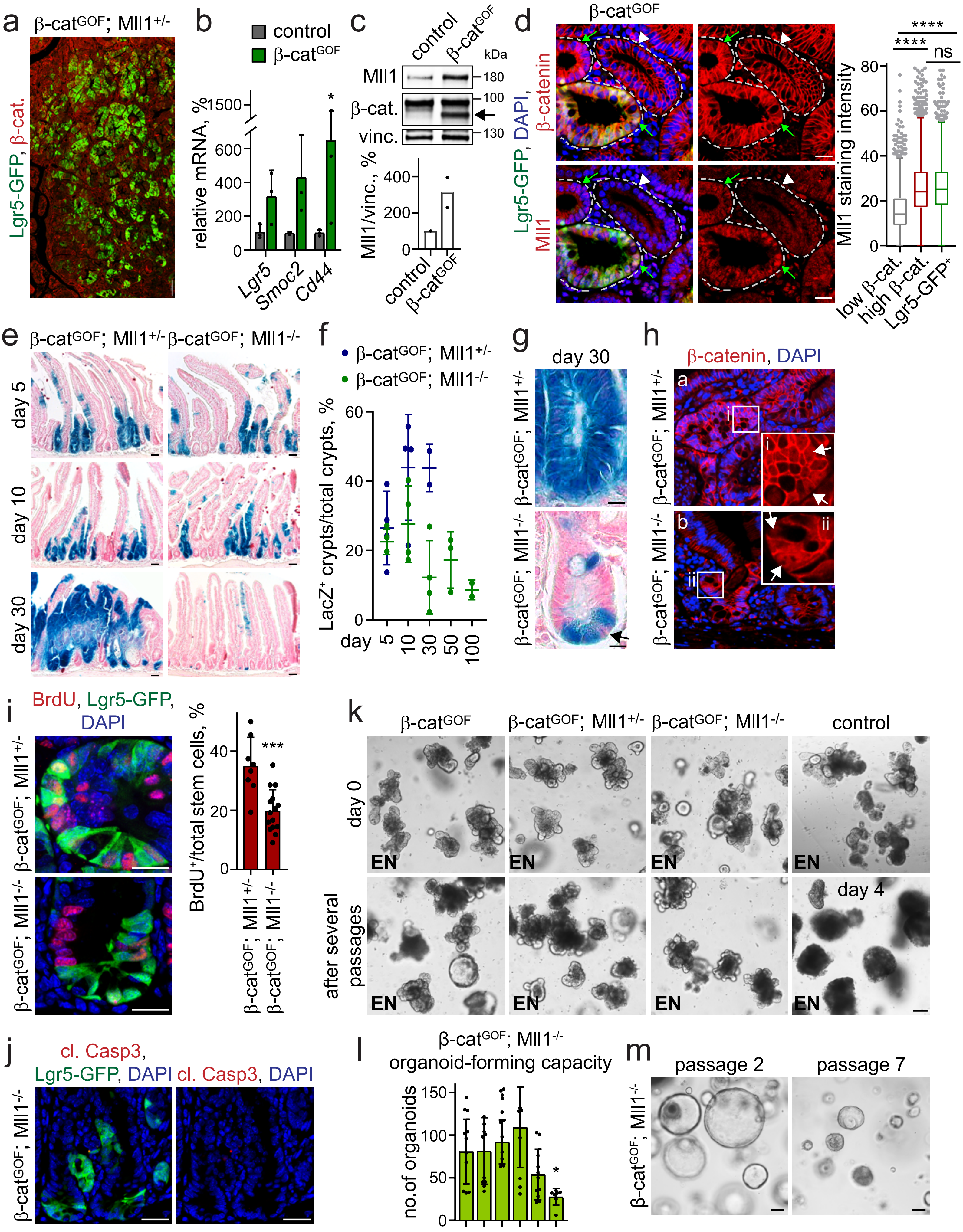

Suppl. Fig. 2 Grinat et al. 2020

Supplementary Fig. 2. **β-catenin^{GOF}-induced intestinal tumorigenesis depends on Mll1.**

a) Tile scan of immunostaining for Lgr5-GFP (green) and β-catenin (red) on β-cat^{GOF}; Mll1$^{+/-}$ mouse tumor section illustrates Lgr5-GFP^{+} stem cell expansion, scale bar 100µm. Stainings were performed in three independent mice. **b)** mRNA expression of the intestinal stem cell markers *Lgr5*, *Smoc2* and *Cd44* in mouse control and β-cat^{GOF} intestines, n=3 independent animals, two-tailed unpaired t test, *p=0.02. Data are presented as mean values ± SD. **c)** Western blot for Mll1 and β-catenin in control and β-cat^{GOF} mouse intestines. Quantification below, normalized to vinculin, n=2 independent mice. Mutant deltaEx3 β-cat^{GOF} marked by arrow. **d)** Immunostaining for β-catenin (red, upper panel), Mll1 (red, lower panel) and Lgr5-GFP (green) on β-cat^{GOF} mouse intestinal tumor section, scale bar 20µm. Green arrows mark β-catenin^{high} cells, white arrowhead β-catenin^{low} cells. Right: quantification of Mll1 staining intensity in low β-catenin, high β-catenin and Lgr5-GFP^{+} tumor cells, n=3, Mann-Whitney test (two-tailed), ****p<0.0001. Box plot indicates median (middle line) and 25th, 75th percentile (box) with Tukey whiskers. **e)** A Rosa-LacZ reporter allele was used to trace recombined cells and their progeny: mutant mouse intestinal cells (blue) in β-cat^{GOF}; Mll1$^{+/-}$ (left panel) and β-cat^{GOF}; Mll1$^{-/-}$ mice (right panel) at day 5 (n=4 β-cat^{GOF}; Mll1$^{+/-}$, n=4 β-cat^{GOF}; Mll1$^{-/-}$), 10 (n=4 β-cat^{GOF}; Mll1$^{+/-}$, n=4 β-cat^{GOF}; Mll1$^{-/-}$), and 30 (n=2 β-cat^{GOF}; Mll1$^{+/-}$, n=4 β-cat^{GOF}; Mll1$^{-/-}$) after induction of mutagenesis, scale bars 50µm. **f)** Quantification of LacZ^{+} crypts per total crypts in β-cat^{GOF}; Mll1$^{+/-}$ and β-cat^{GOF}; Mll1$^{-/-}$ mice at day 5 (n=4 β-cat^{GOF}; Mll1$^{+/-}$, n=4 β-cat^{GOF}; Mll1$^{-/-}$), 10 (n=4 β-cat^{GOF}; Mll1$^{+/-}$, n=4 β-cat^{GOF}; Mll1$^{-/-}$), 30 (n=2 β-cat^{GOF}; Mll1$^{+/-}$, n=4 β-cat^{GOF}; Mll1$^{-/-}$), 50 (n=3 β-cat^{GOF}; Mll1$^{-/-}$) and 100 (n=2 β-cat^{GOF}; Mll1$^{-/-}$), dot plot with mean and standard deviation. β-cat^{GOF}; Mll1$^{+/-}$ mice do not survive past 40 days after induction. **g)** Magnification of LacZ^{+} crypts in β-cat^{GOF}; Mll1$^{+/-}$ (upper panel) and β-cat^{GOF}; Mll1$^{-/-}$ mice (lower panel) at day 30 after induction. Arrow marks non-mutated (LacZ^{-}) stem cell adjacent to long-lived mutant (LacZ^{+}) Paneth cells in crypt of Rosa-LacZ; β-cat^{GOF}; Mll1$^{-/-}$ mice. Paneth cells identified by lysosomal vesicles. Nuclei visualized by nuclear fast red, scale bar 20µm. Representative crypts from two independent β-cat^{GOF}; Mll1$^{+/-}$and 4 independent β-cat^{GOF}; Mll1$^{-/-}$ mice. **h)** Insets a and b of Fig. 2c: β-catenin (red) immunostaining on β-cat^{GOF}; Mll1$^{+/-}$ and β-cat^{GOF}; Mll1$^{-/-}$

intestine, scale bar 20µm. Nuclei in blue (DAPI). Magnifications in insets i and ii, scale bar 10µm. Nuclear β-catenin marked by white arrows. Stainings were performed on sections of three independent mice. **i)** Immunofluorescence for BrdU (red) and Lgr5-GFP (green) in crypts of β-cat^{GOF}; Mll1$^{+/-}$ (upper panel) and β-cat^{GOF}; Mll1$^{-/-}$ mice (lower panel) at day 30 after induction, scale bar 20µm. BrdU injected i.p. 2h before sacrifice. Quantification of BrdU^{+} stem cells to total Lgr5-GFP^{+} stem cells on the right, n=5 independent mice, two-tailed unpaired t test, ***p=0.0003. Data are presented as mean values ± SD. **j)** Immunostaining for cleaved Caspase-3 (red) and Lgr5-GFP (green) on β-cat^{GOF}; Mll1$^{-/-}$ intestine, scale bar 50µm. Stainings were performed in three independent mice. **k)** Brightfield images of tamoxifen-induced Lgr5-GFP-IRES-Cre^{ERT2}; β-cat^{GOF}, Lgr5-GFP-IRES-Cre^{ERT2}; β-cat^{GOF}; Mll1$^{+/-}$ and Lgr5-GFP-IRES-Cre^{ERT2}; β-cat^{GOF}; Mll1$^{-/-}$ intestinal organoids at day 0 and after 4 weeks of R-spondin1-free culture in crypt medium supplemented with EGF and Noggin (EN). Non-induced control organoids do not survive past day 4 in the absence of R-spondin1, scale bar 100µm. Representative images from two independent experiments. **l)** Serial re-plating of β-cat^{GOF}; Mll1$^{-/-}$ intestinal organoids by single cell dissociation. Numbers of organoids counted as triplicates in n=4 independent assays from two biologically independent organoid lines. Cells were cultured in crypt medium supplemented with EGF, Noggin and R-spondin1. Significance calculated versus passage 1, two-tailed unpaired t test, *p=0.013. Data are presented as mean values ± SD. **m)** Brightfield images of single cell-derived β-cat^{GOF}; Mll1$^{-/-}$ organoids at passages 2 and 7, scale bars 100µm. Representative images from 4 independent experiments with two biologically independent organoid lines. Source data are provided as a Source Data file.

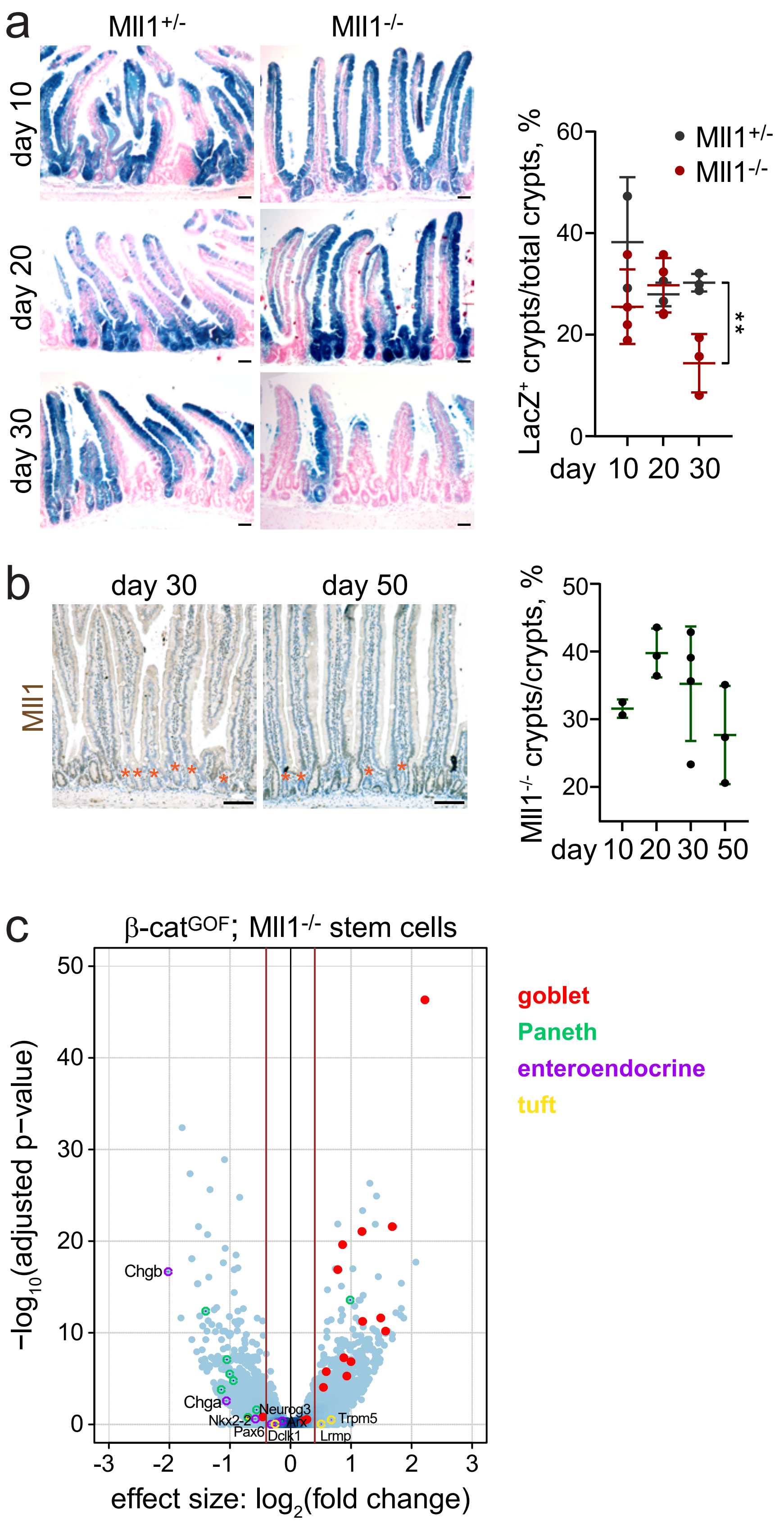

Suppl. Fig. 3 Grinat et al. 2020

Supplementary Fig. 3. **Lineage tracing of Mll1-deficient Lgr5^{+} intestinal stem cells.**

a) Lineage tracing: sections of LacZ (blue) whole-mount stainings of intestines of Lgr5-EGFP-IRES-Cre^{ERT2}; Rosa-LacZ; Mll1$^{flox/+}$ and Lgr5-EGFP-IRES-Cre^{ERT2}; Rosa-LacZ; Mll1$^{flox/flox}$ mice (referred to as Mll1$^{+/-}$ and Mll1$^{-/-}$, respectively) at day 10 (n=2 Mll1$^{+/-}$, n=4 Mll1$^{-/-}$), 20 (n=3 Mll1$^{+/-}$, n=5 Mll1$^{-/-}$) and 30 (n=3 Mll1$^{+/-}$, n=3 Mll1$^{-/-}$) after Cre induction. Nuclear fast red counterstaining, scale bar 50µm. Right: Quantification of LacZ^{+} crypts per total crypts, dot plot with mean ± SD, two-tailed unpaired t test, **p=0.006. **b)** Immunohistochemistry for Mll1 on sections of Mll1$^{-/-}$ mouse intestines at days 30 and 50 after induction, scale bars 100µm. Orange asterisks mark Mll1 knockout crypts. Quantification of Mll1 knockout crypts per total crypts days 10 (n=2), 20 (n=3), 30 (n=4) and 50 (n=3) after induction on the right, dot plot with mean and standard deviation. **c)** Volcano plot of differentially expressed genes in β-cat^{GOF}; Mll1$^{-/-}$ relative to β-cat^{GOF}; Mll1$^{+/-}$ Lgr5-GFP^{+} stem cells isolated from 4 independent mice each, represented as blue dots. Cut-off $\log_2$ fold change ≥ 0.5. Goblet (red), Paneth (green), enteroendocrine (purple) and tuft (yellow) cell-specific genes (Haber et al. 2017, Nature) are marked. Enteroendocrine and tuft cell genes are indicated by names. Source data are provided as a Source Data file.

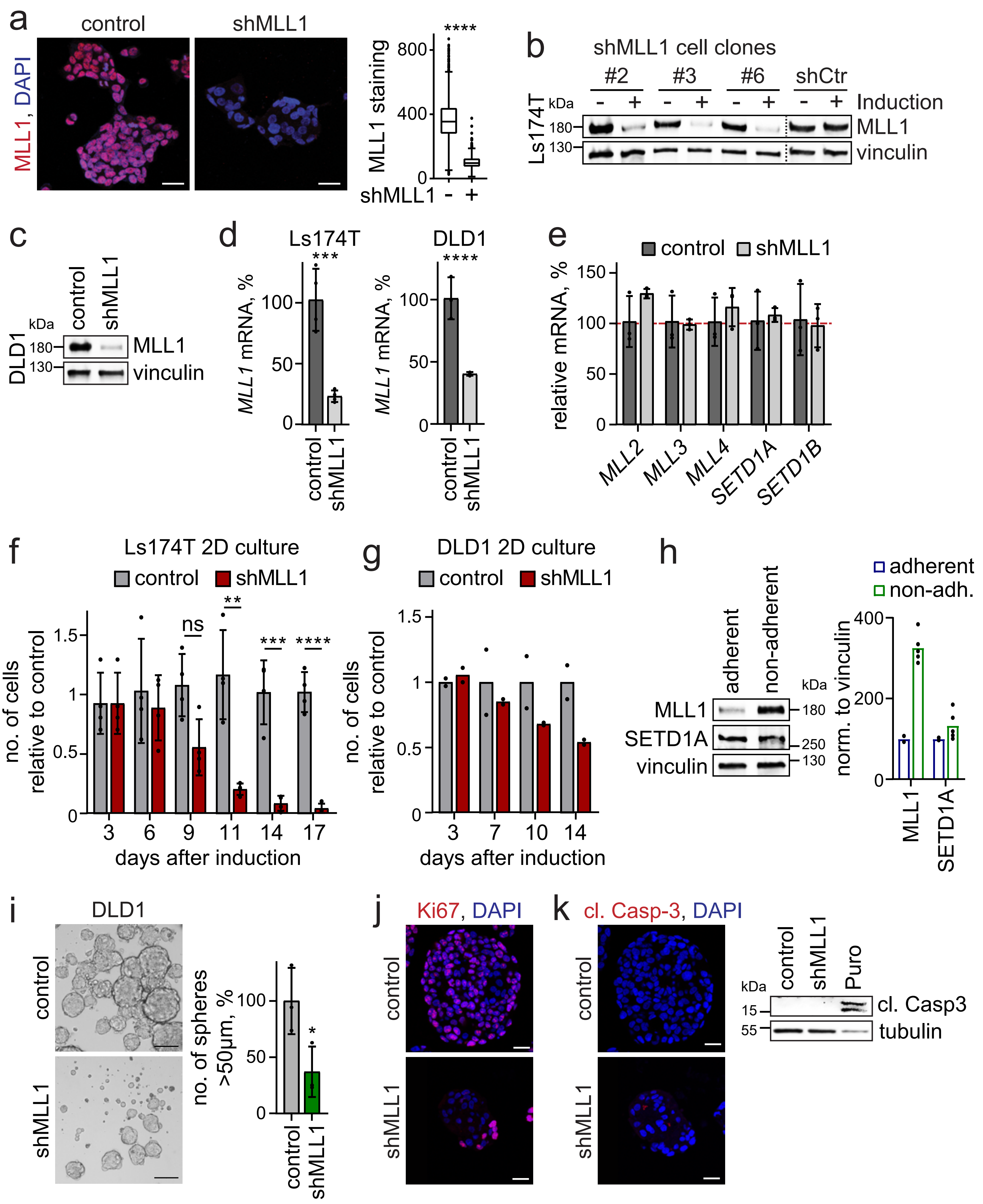
a
control
shMLL1
MLL1, DAPI
MLL1 staining
****
800
400
0
shMLL1 - +
b
shMLL1 cell clones
#2
#3
#6
shCtr
Ls174T
kDa
- + - + - + - +
Induction
180
130
MLL1
vinculin
c
DLD1
kDa
control
shMLL1
180
130
MLL1
vinculin
d
Ls174T
DLD1
***
****
MLL1 mRNA, %
100
50
0
control
shMLL1
e
control
shMLL1
relative mRNA, %
150
100
50
0
MLL2
MLL3
MLL4
SETD1A
SETD1B
f
Ls174T 2D culture
control
shMLL1
no. of cells relative to control
1.5
1
0.5
0
ns
**
***
****
3 6 9 11 14 17
days after induction
g
DLD1 2D culture
control
shMLL1
no. of cells relative to control
1.5
1
0.5
0
3 7 10 14
days after induction
h
adherent
non-adherent
kDa
MLL1
SETD1A
vinculin
180
250
130
adherent
non-adh.
norm. to vinculin
400
200
0
MLL1
SETD1A
i
DLD1
control
shMLL1
no. of spheres >50μm, %
100
50
0
*
control
shMLL1
j
Ki67, DAPI
control
shMLL1
k
cl. Casp-3, DAPI
control
shMLL1
kDa
control
shMLL1
Puro
15
55
cl. Casp3
tubulin

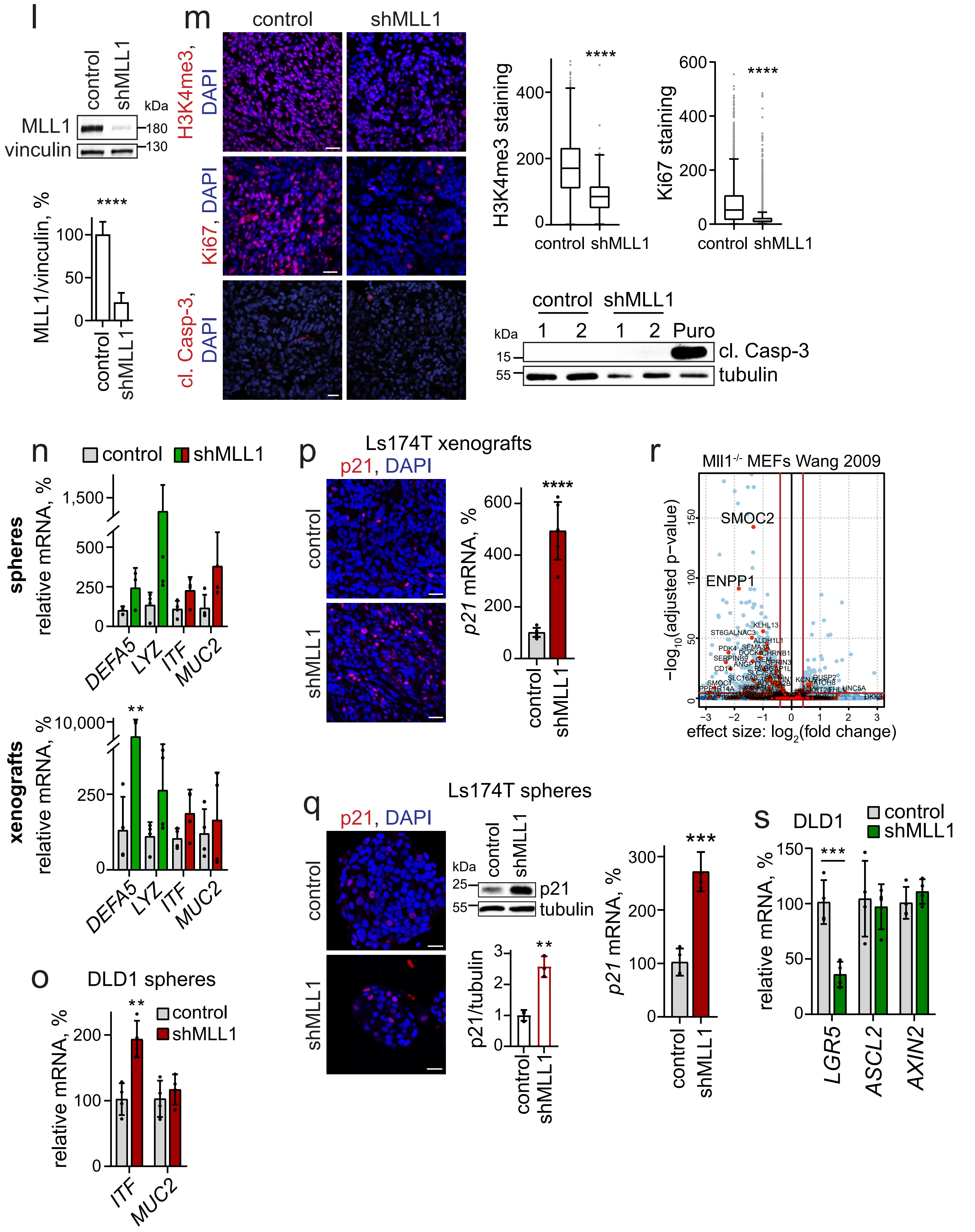

Suppl. Fig. 4_continued Grinat et al. 2020

Supplementary Fig. 4. **Mll1 sustains stemness and prevents differentiation of colon cancer cells.**

a) Immunofluorescence for MLL1 (red) on control and 3d doxycycline-induced shMLL1 human Ls174T cells. Nuclei in blue (DAPI), scale bar 20µm. Right: quantification of MLL1 staining intensity in control and three shMLL1 human Ls174T cell clones, n=7 independent samples, Mann-Whitney test, ****p<0.0001. Box plot indicates median (middle line) and 25th, 75th percentile (box) with Tukey whiskers. **b)** MLL1 Western blot of three human Ls174T shMLL1 cell clones #2, #3, #6 both non-induced and 3d doxycycline-induced, and shCtr cells with shRNA against Renilla luciferase. Representative Western blot from three independent experiments, shMLL1 and shRenilla samples derive from the same experiment and were processed in parallel but run on separate gels. **c)** MLL1 Western blot of non-induced control and 3d doxycycline-induced human DLD1 shMLL1 cells. Vinculin as control for equal loading. Representative Western blot from two independent experiments. **d)** *MLL1* mRNA expression in control and shMLL1 Ls174T, n=4 independent experiments with three biologically independent samples, and DLD1 cells, n=3 independent experiments, two-tailed unpaired t test, ***p=0.0009, ****p<0.0001. Data are presented as mean values ± SD. **e)** mRNA expression of *MLL* family members in shMLL1 and control Ls174T cells, n=4 independent experiments with three biologically independent samples. Data are presented as mean values ± SD. **f, g)** Serial re-plating assay: quantification of viable **(f)** shMLL1 Ls174T and **(g)** shMLL1 DLD1 cells cultured under 2D adherent conditions at indicated time points after shRNA induction, number of cells relative to control cells, n=4 (Ls174T) over two independent experiments, two-tailed unpaired t test, **p=0.002, ***p=0.0005, ****p<0.0001, data are presented as mean values ± SD, n=2 samples (DLD1) over two independent experiments. **h)** Western blot for MLL1 and SETD1A in human Ls174T cells cultured under adherent and non-adherent conditions, normalized to vinculin. Quantification on the right, n=2 adherent and n=5 non-adherent samples from three biologically independent cell clones. **i)** Brightfield images of control and 8d doxycycline-induced shMLL1 human DLD1 spheres, scale bar 100µm. Quantification of spheres >50µm in size on the right, n=3 independent experiments,

two-tailed unpaired t test, *p=0.04. Data are presented as mean values ± SD. **j)** Ki67 staining (red) in control and shMLL1 human Ls174T spheres, scale bar 20µm. Nuclei in blue (DAPI). Stainings were performed in spheres of two biologically independent cell clones. **k)** Left: immunostaining for cleaved Caspase-3 (red) in control and shMLL1 human Ls174T spheres, scale bar 20µm. Nuclei in blue (DAPI). Stainings were performed in spheres of two biologically independent cell clones. Right: Western blot for cleaved Caspase-3, normalized to tubulin. Puromycin (2µg/ml) treatment for 24h was used as control for apoptosis. **l)** Western blot for MLL1 from control and shMLL1 human Ls174T xenografts at day 28, normalized to vinculin, n=6 tumors, two-tailed unpaired t test, ****p<0.0001. Data are presented as mean values ± SD. **m)** Immunofluorescence for H3K4me3 (upper panel), Ki67 (middle panel) and cleaved Caspase-3 (lower panel) on sections of control and shMLL1 xenografts, scale bar 20µm. Nuclei in blue (DAPI). Quantifications on the right, n=4 tumors, Mann-Whitney test (two-tailed), ****p<0.0001. Box plots indicate median (middle line) and 25th, 75th percentile (box) with Tukey whiskers. Cleaved Caspase-3 and tubulin Western blot of two pairs of control and shMLL1 tumors (right). Puromycin (2µg/ml) treatment for 24h was used as control for apoptosis. **n)** mRNA expression of Paneth (green) and goblet cell (red) markers in human shMLL1 Ls174T sphere cells (n=4) and xenograft tumors (n=6) relative to non-induced controls. Two-tailed unpaired t test for significance, **p=007. Data are presented as mean values ± SD. **o)** mRNA expression of goblet cell markers *ITF* and *MUC2* in human shMLL1 DLD1 sphere cells relative to control sphere cells, n=4 biologically independent samples, two-tailed unpaired t test, **p=0.0025. Data are presented as mean values ± SD. **p)** Left: immunostaining for p21 (red) in control and shMLL1 human Ls174T xenografts. Nuclei in blue (DAPI), scale bar 20µm. Right: *p21* mRNA expression in control and shMLL1 xenografts, n=6 tumors, two-tailed unpaired t test, ****p<0.0001. Data are presented as mean values ± SD. **q)** Left: immunostaining for p21 (red) on control and shMLL1 human Ls174T sphere cells. Nuclei in blue (DAPI), scale bar 20µm. Middle: Western blotting for p21. Quantification below, normalized to tubulin, n=3 biologically independent samples, two-tailed unpaired t test, **p=0.002. Right: *p21* mRNA expression in control and shMLL1 human Ls174T sphere cells, n=4 samples derived from

three biologically independent cell clones, two-tailed unpaired t test, ***p=0.0003. Data are presented as mean values ± SD. **r)** Volcano plot of differentially expressed genes in shMLL1 cells, represented as blue dots. Genes from Mll1$^{-/-}$ MEF signature marked in red and indicated by name. **s)** mRNA expression of Wnt-regulated stem cell genes in 8d doxycycline-induced control and shMLL1 human DLD1 sphere cells, n=4 independent experiments, two-tailed unpaired t test, ***p=0.0002. Data are presented as mean values ± SD. Source data are provided as a Source Data file.

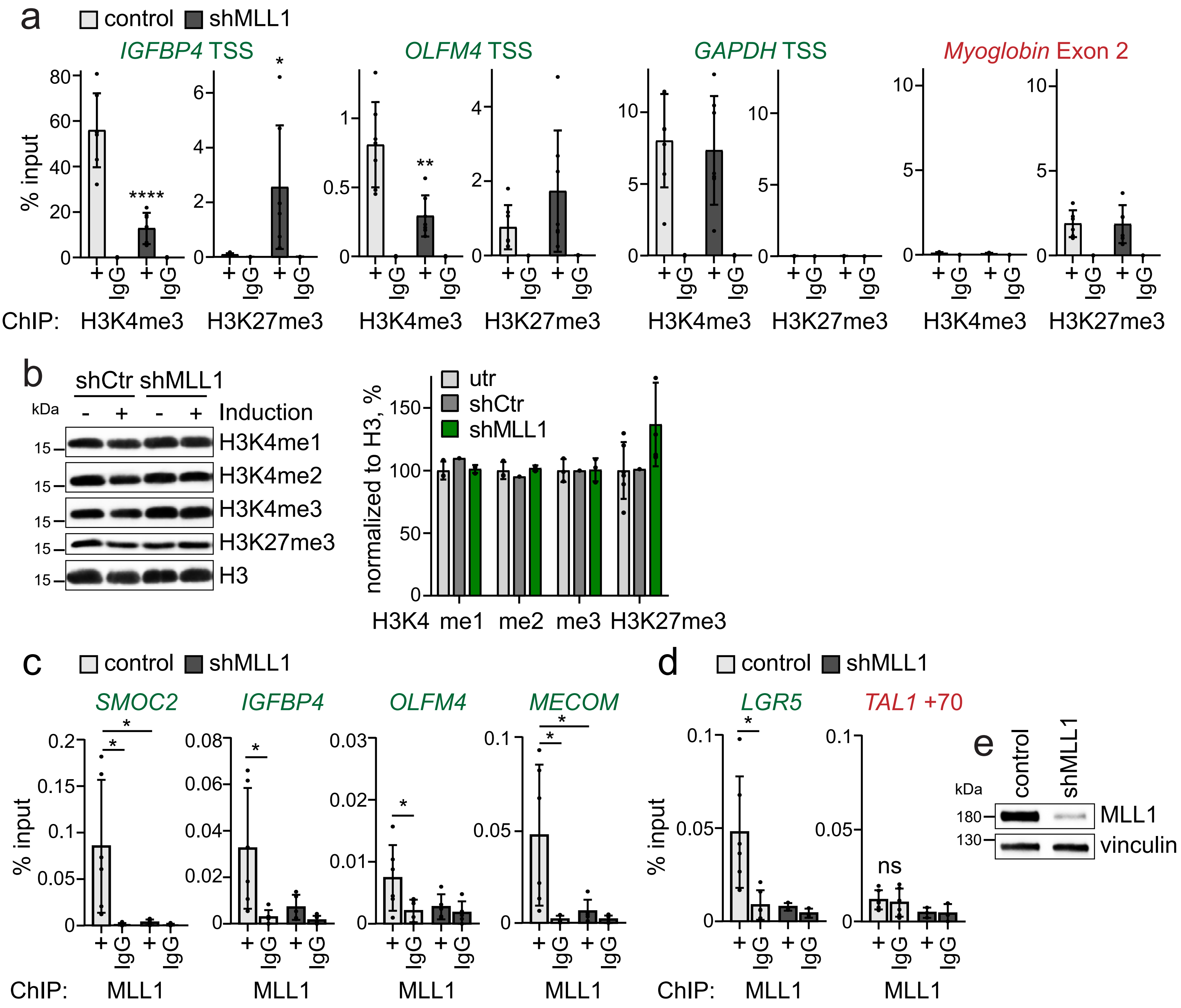

Suppl. Fig. 5 Grinat et al. 2020

Supplementary Fig. 5. **Mll1 regulates specific stem cell genes.**

a) ChIP for H3K4me3 and H3K27me3 at the TSSs of *IGFBP4* and *OLFM4* in non-induced control (light grey columns) and 11d doxycycline-induced shMLL1 Ls174T cells (black columns), represented as % input, n=7 samples derived from two biologically independent cell clones over 4 independent experiments, two-tailed unpaired t test, significance calculated for control versus shMLL1, *IGFBP4* TSS ****p<0.0001, *p=0.013; *OLFM4* TSS **p=0.0018. *GAPDH* TSS and *Myoglobin* Exon 2 positive and negative controls. Data are presented as mean values ± SD. **b)** Western blot for global H3K4 and H3K27 methylation in non-induced (utr) and 6d doxycycline-induced shCtr and shMLL1 cells. Samples derive from the same experiment, samples for H3K4 modification blots were prepared as a mastermix to be loaded on separate gels and processed in parallel. Quantification on the right, normalized to total H3, n=3 biologically independent samples. Data are presented as mean values ± SD. **c)** ChIP for MLL1 in non-induced control (light grey columns) and 6d doxycycline-induced shMLL1 Ls174T cells (black columns), binding at the *SMOC2*, *IGFBP4* and *OLFM4* promoters, represented as % input, n=6 control and n=5 shMLL1 samples derived from two biologically independent cell clones over 4 independent experiments, two-tailed unpaired t test, significance calculated for control versus shMLL1 and IgG *SMOC2* *p=0.032, *p=0.016; *IGFBP4* *p=0.019; *OLFM4* *p=0.042; *MECOM* *p=0.042, *p=0.015. *MECOM* TSS positive control. Data are presented as mean values ± SD. **d)** ChIP for MLL1 in non-induced control (n=6 samples over three independent experiments, light grey columns) and 8d doxycycline-induced shMLL1 human DLD1 cells (n=3 independent experiments, black columns), binding at the *LGR5* promoter, negative control region *TAL1* +70, represented as % input, two-tailed unpaired t test, significance calculated for control versus IgG, *p=0.011. Data are presented as mean values ± SD. **e)** Western blot for MLL1 in cells used for ChIP analyses, representative blot from three independent experiments. Source data are provided as a Source Data file.

Supplementary Table 1: Primer sequences for ChIP-qPCR.

ChIP-qPCR primer	sequence (5'-3')
ASCL2 fwd	GATCCGCCAGCACTAGAGAC
ASCL2 rev	TGGCTTCGATTTCGCTCACT
AXIN2 fwd	ACTAAGAAGGGTAGGGGCTGAA
AXIN2 rev	CTCGAGGCTCTGGTCGGG
AXIN2_TCF4 fwd	GGCTTTCTTTGAAGCGGCTC
AXIN2_TCF4 rev	TAACCCCTCAGAGCGATGGA
IGFBP4 fwd	GAAGGCAGGTACGTGGCAG
IGFBP4 rev	CACTGGGATTTCACCCTCGG
IGFBP4_Mll1 fwd	TGCACACACTGATGCACGG
IGFBP4_Mll1 rev	CACTCAGGAGGGAATTGGGA
LGR5 fwd	GAGGGGAAGGAAGGTTCGTC
LGR5 rev	CATTTCTCTGCGGGTCCAGA
LGR5_Mll1 fwd	CTACTTCGGGCACCATGGAC
LGR5_Mll1 rev	CTCAGCAACACACCAGACCT
LGR5_TCF4 fwd	GCAACCACAAACCCCGATTA
LGR5_TCF4 rev	CCCGGGGGAAGAGATGGTTA
MECOM fwd	GAAACCGACGGACAGAGACA
MECOM rev	TCCTTGTTCCTCCTGCGAAA
OLFM4 fwd	ACTCAGATTCCTGGGTGTCCT
OLFM4 rev	TGTCCTCTTAGCTGGAGCCG
OLFM4_Mll1 fwd	GGGAAGAAGGCAGAGGTCAC
OLFM4_Mll1 rev	CCTGAGCTTCTTGTGAGCCA
SMOC2 fwd	CCTGTGGGGAACCAGGTCT
SMOC2 rev	CGGCTCACTTACGAGGGTC
SMOC2_Mll1 fwd	GACCTGGAGCTCAAGTGGTG
SMOC2_Mll1 rev	TCCTACCTGCTCCAGCCTC
TAL1 +70 fwd[1]	GTGGCCACAAAGCAAGGAAT
TAL1 +70 rev[1]	TCTCTGGAATCTCCAAGGCAA

Supplementary Table 2: Primer sequences for qRT-PCR.

RT-PCR primer	species	sequence (5'-3')
Ascl2_fwd[2]	mouse	AAAGCTTGGTCCGGTTCTTCATCC
Ascl2_rev[2]	mouse	GCAGATGCTTAGCTTATTGCGTCC
Axin2_fwd	mouse	AGTCAGCAGAGGGACAGGAA
Axin2_rev	mouse	CTTCGTACATGGGGAGCACT
Bmp4_fwd	mouse	AGAAGAATAAGAACTGCCGTCGCC
Bmp4_rev	mouse	ATGGCATGGTTGGTTGAGTTGAGG

Cd44_fwd[2]	mouse	CACCTTGGCCACCACTCCTA
Cd44_rev[2]	mouse	TTGGATGTGAGATTGGGTCGAA
Gapdh_fwd[2]	mouse	AAATGGTGAAGGTCGGTGTGAACG
Gapdh_rev[2]	mouse	TGATGACAAGCTTCCCATTCTCGG
Gata4_fwd	mouse	AAACGGAAGCCCAAGAACCT
Gata4_rev	mouse	ACACAGTACTGAATGTCTGGGA
Lgr5_fwd[3]	mouse	CCTACTCGAAGACTTACCCAGT
Lgr5_rev[3]	mouse	GCATTGGGGTGAATGATAGCA
Mmp7_fwd	mouse	AGGAGTGAACTTCCTGTTTGCTGC
Mmp7_rev	mouse	TTCTGAATGCCTGCAATGTCGTCC
Muc2_fwd[2]	mouse	TGTGATGCCAATGACAAGGTGTCC
Muc2_rev[2]	mouse	ACCACAATGTTGATGCCAGACTCG
Olfm4_fwd[2]	mouse	TTGGGTTACCAAGCAGTACAAGCC
Olfm4_rev[2]	mouse	TCTCATTGTGATGCCTAGGTTGCC
Smoc2_fwd[2]	mouse	CATAGGACCGCGGAGCAGTT
Smoc2_rev[2]	mouse	AGGCTGCAGTCTCTGTCTTTG
ASCL2_fwd	human	GCGTGAAGCTGGTGAACTTG
ASCL2_rev	human	GGATGTACTCCACGGCTGAG
AXIN2_fwd	human	AACTGAAACTGGAGCTGGAAAGCC
AXIN2_rev	human	TTTGTGGGTCCTCTTCATAGCTGC
CD44_fwd	human	GGCTTTCAATAGCACCTTGC
CD44_rev	human	ACACCCCTGTGTTGTTTGCT
DEFA5_fwd	human	TCCCTCCTGCAGGTGACCCCA
DEFA5_rev	human	GTGGCTCTTGCCTGAGAACCTGA
GAPDH_fwd	human	AAGGTGAAGGTCGGAGTCAA
GAPDH_rev	human	AATGAAGGGGTCATTGATGG
IGFBP4_fwd	human	TTCCACCCCAAGCAGTGTCA
IGFBP4_rev	human	TCCAGAGCACAGGAGTAGCA
ITF_fwd[2]	human	ATCCAGAGCAGCTGTGCAAACAAC
ITF_rev[2]	human	TTTGCAGACAGGCCCACGTACTC
LGR5_fwd	human	TGCTTACCAGTGCTGTGCATTTGG
LGR5_rev	human	TGCACTGAATGAAGGGCTTTCAGG
LRIG1_fwd	human	GGTGAGCCTGGCCTTATGTGAATA
LRIG1_rev	human	CACCACCATCCTGCACCTCC
LYZ_fwd[2]	human	GCCTAGCAAACTGGATGTGTTTGG
LYZ_rev[2]	human	TTTGCACAAGCTACAGCATCAGCG
MLL1 (KMT2A)_fwd	human, mouse	TGAGCTGCAGATGACTGGTTA

MLL1 (KMT2A)_rev	human, mouse	CCAGAGCATCAGAGGAGAGC
MLL2 (KMT2B)_fwd	human	ATCCCAGCGAGGTCGAG
MLL2 (KMT2B)_rev	human	GCCGGAGAAGTTCAGTCAGT
MLL3 (KMT2C)_fwd	human	CAAAGAACAATCTGCAGAAGAGGA
MLL3 (KMT2C)_rev	human	GGTTGGTTTCTCCATGGCAAG
MLL4 (KMT2D)_fwd	human	CCCTGAGTATCTGAAGGGCG
MLL4 (KMT2D)_rev	human	AGGGTATGGGGCCGTTTGTA
MUC2_fwd[2]	human	TTTGATGCCAGCATTTGCATCCCG
MUC2_rev[2]	human	TTGGCCGAGTACATGACAAATGTCCC
OLFM4_fwd	human	ATCATCTGCCTCTTCAGGCG
OLFM4_rev	human	CCCAGGTTTCTTCCAGGCAT
p21_fwd	human	AGTCAGTTCCTTGTGGAGCC
p21_rev	human	CATTAGCGCATCACAGTCGC
SETD1A_fwd	human	GTCGAAGACCTCCAAGACCC
SETD1A_rev	human	ACCTCTTCCACCTCACCGTA
SETD1B_fwd	human	ACTCGTTGGGCATGGAAGAG
SETD1B_rev	human	GGCTCTGGAGACAGCAACAT
SMOC2_fwd	human	AAGCCCGGAAGGAGTTTCAG
SMOC2_rev	human	TGCGGCATCATCTGTTTTTCC
SOX9_fwd	human	AGTACCCGCACTTGCACAAC
SOX9_rev	human	CGTTCTTCACCGACTTCCTC

Supplementary Table 3: Human CRC transcriptomic datasets.

dataset	N.dataset (stage available)	reference	GEO
TCGA-COAD	393 (383)	4	https://www.cancer.gov/about-nci/organization/ccg/research/structural-genomics/tcga
TCGA-READ	150 (142)	4	https://www.cancer.gov/about-nci/organization/ccg/research/structural-genomics/tcga
GSE14333	290	5	https://www.ncbi.nlm.nih.gov/gds/?term=gse14333
GSE33113	90	6	https://www.ncbi.nlm.nih.gov/gds/?term=gse33113
GSE39582	562	7	https://www.ncbi.nlm.nih.gov/gds/?term=gse39582
GSE38832	122	8	https://www.ncbi.nlm.nih.gov/gds/?term=gse38832
GSE44076	98	9	https://www.ncbi.nlm.nih.gov/gds/?term=gse44076
GSE13294	155 (147)	10	https://www.ncbi.nlm.nih.gov/gds/?term=gse13294
GSE18088	53	11	https://www.ncbi.nlm.nih.gov/gds/?term=gse18088
GSE2109	320	-	https://www.ncbi.nlm.nih.gov/gds/?term=gse2109
**Total**	2233 (2207)		

Supplementary References

1. Salz, T. *et al.* hSETD1A regulates Wnt target genes and controls tumor growth of colorectal cancer cells. *Cancer Res* **74**, 775-786 (2014).
2. Heuberger, J. *et al.* Shp2/MAPK signaling controls goblet/paneth cell fate decisions in the intestine. *Proc Natl Acad Sci U S A* **111**, 3472-3477 (2014).
3. Garcia, M.I. *et al.* LGR5 deficiency deregulates Wnt signaling and leads to precocious Paneth cell differentiation in the fetal intestine. *Dev Biol* **331**, 58-67 (2009).
4. Liu Y., S.N.S., Hinoue T., et al. Comparative Molecular Analysis of Gastrointestinal Adenocarcinomas. *Cancer Cell* **33**, 721-735 (2018).
5. Jorissen, R.N. *et al.* Metastasis-Associated Gene Expression Changes Predict Poor Outcomes in Patients with Dukes Stage B and C Colorectal Cancer. *Clin Cancer Res* **15**, 7642-7651 (2009).
6. Kemper, K. *et al.* Mutations in the Ras-Raf Axis underlie the prognostic value of CD133 in colorectal cancer. *Clin Cancer Res* **18**, 3132-3141 (2012).
7. Marisa, L. *et al.* Gene expression classification of colon cancer into molecular subtypes: characterization, validation, and prognostic value. *PLoS Med* **10**, e1001453 (2013).
8. Tripathi, M.K. *et al.* Nuclear factor of activated T-cell activity is associated with metastatic capacity in colon cancer. *Cancer Res* **74**, 6947-6957 (2014).
9. Jorissen, R.N. *et al.* DNA copy-number alterations underlie gene expression differences between microsatellite stable and unstable colorectal cancers. *Clin Cancer Res* **14**, 8061-8069 (2008).
10. Sanz-Pamplona, R. *et al.* Aberrant gene expression in mucosa adjacent to tumor reveals a molecular crosstalk in colon cancer. *Mol Cancer* **13**, 46 (2014).
11. Grone, J. *et al.* Molecular profiles and clinical outcome of stage UICC II colon cancer patients. *Int J Colorectal Dis* **26**, 847-858 (2011).
