# Supplementary material for: The epigenetic regulator Mll1 is required for Wnt-driven intestinal tumorigenesis and cancer stemness
Source: Nat Commun. 2020 Dec 21;11:6422. doi: 10.1038/s41467-020-20222-z (PMC7752919; doi:10.1038/s41467-020-20222-z)
Supplement: Supplementary file 3 — Description of Additional Supplementary Files [file 41467_2020_20222_MOESM3_ESM.pdf]

Description of Additional Supplementary Files

Supplementary Data 1: **Differentially expressed genes in intestinal stem cells from β-cat^{GOF}; Mll1$^{+/-}$ and β-cat^{GOF}; Mll1$^{-/-}$ mice.** Differential gene expression analysis was performed with DESeq2, for which a maximum of 10% false discoveries (FDR 10) was accepted. Genes with adjusted p-value lower than 0.05 and absolute fold-change (log10) higher than 0.5 were considered differentially expressed.

Supplementary Data 2: Differentially expressed genes in shMLL1 Ls174T colon cancer cells. Comparisons between shMLL1 and parental control cells were corrected by individual technical replicates (paired comparison). In order to avoid background signal/noise, genes with less than 10 reads over all samples were excluded before adjusting the p-value for multiple testing. Genes with adjusted p-value lower than 0.05 and absolute fold-change (log10) higher than 0.5 were considered differentially expressed.

Supplementary Data 3: MLL1-regulated colon cancer stem cell signature. Differentially expressed genes in shMLL1 Ls174T colon cancer cells were compared to three previously established intestinal stem cell signatures. All genes that were part of at least one of the signatures were compiled to establish the MLL1-regulated colon cancer stem cell signature.
