# Supplementary material for: The epigenetic regulator Mll1 is required for Wnt-driven intestinal tumorigenesis and cancer stemness
Source: Nat Commun. 2020 Dec 21;11:6422. doi: 10.1038/s41467-020-20222-z (PMC7752919; doi:10.1038/s41467-020-20222-z)
Supplement: Supplementary file 7 — Reporting summary [file 41467_2020_20222_MOESM7_ESM.pdf]

nature research

Corresponding author(s): Walter Birchmeier, Julian Heuberger

Last updated by author(s): Oct 16, 2020

Reporting Summary

Nature Research wishes to improve the reproducibility of the work that we publish. This form provides structure for consistency and transparency in reporting. For further information on Nature Research policies, see our Editorial Policies and the Editorial Policy Checklist.

Statistics

For all statistical analyses, confirm that the following items are present in the figure legend, table legend, main text, or Methods section.

n/a	Confirmed	
☐	☒	The exact sample size (n) for each experimental group/condition, given as a discrete number and unit of measurement
☐	☒	A statement on whether measurements were taken from distinct samples or whether the same sample was measured repeatedly
☐	☒	The statistical test(s) used AND whether they are one- or two-sided<br>*Only common tests should be described solely by name; describe more complex techniques in the Methods section.*
☒	☐	A description of all covariates tested
☐	☒	A description of any assumptions or corrections, such as tests of normality and adjustment for multiple comparisons
☐	☒	A full description of the statistical parameters including central tendency (e.g. means) or other basic estimates (e.g. regression coefficient) AND variation (e.g. standard deviation) or associated estimates of uncertainty (e.g. confidence intervals)
☐	☒	For null hypothesis testing, the test statistic (e.g. F, t, r) with confidence intervals, effect sizes, degrees of freedom and P value noted<br>*Give P values as exact values whenever suitable.*
☒	☐	For Bayesian analysis, information on the choice of priors and Markov chain Monte Carlo settings
☒	☐	For hierarchical and complex designs, identification of the appropriate level for tests and full reporting of outcomes
☒	☐	Estimates of effect sizes (e.g. Cohen's d, Pearson's r), indicating how they were calculated

Our web collection on statistics for biologists contains articles on many of the points above.

Software and code

Policy information about availability of computer code

Data collection	FASTQC Andrews S. (2010). FastQC: a quality control tool for high throughput sequence data. Available online at: http://www.bioinformatics.babraham.ac.uk/projects/fastqc<br>STAR aligner https://www.ncbi.nlm.nih.gov/pubmed/23104886<br>DESeq2 RRID:SCR_015687; https://genomebiology.biomedcentral.com/articles/10.1186/s13059-014-0550-8<br>featureCounts software (version 1.6.3) Liao Y, Smyth GK and Shi W. featureCounts: an efficient general purpose program for assigning sequence reads to genomic features. Bioinformatics, 30(7):923-30, 2014<br>GSNAP (version 2018-07-04)<br>Zeiss ZEN (blue edition)<br>Leica Application Suite X (LAS X)
Data analysis	GraphPad Prism 7, Fiji ImageJ (https://fiji.sc/), Imaris 8 (Bitplane/Andor), R Rv3.4

For manuscripts utilizing custom algorithms or software that are central to the research but not yet described in published literature, software must be made available to editors and reviewers. We strongly encourage code deposition in a community repository (e.g. GitHub). See the Nature Research guidelines for submitting code & software for further information.

Data

Policy information about availability of data

All manuscripts must include a data availability statement. This statement should provide the following information, where applicable:

- Accession codes, unique identifiers, or web links for publicly available datasets
- A list of figures that have associated raw data
- A description of any restrictions on data availability

The RNA sequencing data from FACS-isolated Lgr5-GFP+ intestinal stem cells (Fig. 3b, d, Supplementary Fig. 3c, Supplementary Data 1) have been deposited in the Gene expression omnibus under accession number GSE148394 [https://www.ncbi.nlm.nih.gov/geo/query/acc.cgi?acc=GSE148394]. The RNA sequencing data from shMLL1 Ls174T colon cancer cells (Fig. 4c, d, Supplementary Fig. 4r, Supplementary Data 2) are available in the ArrayExpress database under accession number E-MTAB-8152 [https://www.ebi.ac.uk/arrayexpress/experiments/E-MTAB-8152/]. The human colon cancer data referenced during the study are available in public repositories from the TCGA website (TCGA-COAD [https://www.cancer.gov/about-nci/organization/ccg/research/structural-genomics/tcga], TCGA-READ [https://www.cancer.gov/about-nci/organization/ccg/research/structural-genomics/tcga]) and in the Gene Expression Omnibus (GEO) at NCBI (GSE14333 [https://www.ncbi.nlm.nih.gov/gds/?term=gse14333], GSE33113 [https://www.ncbi.nlm.nih.gov/gds/?term=gse33113], GSE39582 [https://www.ncbi.nlm.nih.gov/gds/?term=gse39582], GSE38832 [https://www.ncbi.nlm.nih.gov/gds/?term=gse38832], GSE44076 [https://www.ncbi.nlm.nih.gov/gds/?term=gse44076], GSE13294 [https://www.ncbi.nlm.nih.gov/gds/?term=gse13294], GSE18088 [https://www.ncbi.nlm.nih.gov/gds/?term=gse18088] and GSE2109 [https://www.ncbi.nlm.nih.gov/gds/?term=gse2109]). Clinical information was obtained from Synapse ID syn2623706 [https://www.synapse.org/#!Synapse:syn2623706/wiki/s]. The source data underlying Figures 1b, 1f, 1g, 2b, 2f, 2g, 3a, 3f, 4a, 4b, 4e, 4f, 5a-d, 6a-d in the main manuscript and Supplementary Figures 1a, 1e, 2b-d, 2f, 2i, 2l, 3a-b, 4a-i, 4k, 4m-q, 4s, 5a-e are provided as a Source Data file. All the other data supporting the findings of this study areis available within the Articlearticle and its, SSupplementary Information files and Source Data, or and available from the corresponding authors upon reasonable request. Source data are provided with this paper.

Field-specific reporting

Please select the one below that is the best fit for your research. If you are not sure, read the appropriate sections before making your selection.

- [x] Life sciences
- [ ] Behavioural & social sciences
- [ ] Ecological, evolutionary & environmental sciences

For a reference copy of the document with all sections, see nature.com/documents/nr-reporting-summary-flat.pdf

Life sciences study design

All studies must disclose on these points even when the disclosure is negative.

Sample size	No sample size calculation was performed. Sample sizes were chosen based on previous published work using similar methods (Wend et al. 2013, EMBO J; Zhu et al. 2019, Cell Reports; Heuberger et al. 2014, PNAS) and on preliminary data from our laboratory.
Data exclusions	No data were excluded.
Replication	All experiments were performed from several independent biological samples across different days. Replication of in vivo studies was achieved by using a large sample size: n=24 bCatGOF; Mll1+/- and n=32 bCatGOF; Mll1-/- independent mice in more than 10 independent experiments. Replication of in vitro studies was achieved by repeating each experiment independently on separate occasions as indicated in the figure legends. Some experiments were performed by independent researchers. All findings were successfully reproduced.
Randomization	All allocations were random.
Blinding	Investigators were not blinded, as phenotypic observations were strong and clearly seen. Although mouse genotypes were known, data collection and analyses were performed unbiasedly.

Reporting for specific materials, systems and methods

We require information from authors about some types of materials, experimental systems and methods used in many studies. Here, indicate whether each material, system or method listed is relevant to your study. If you are not sure if a list item applies to your research, read the appropriate section before selecting a response.

Materials & experimental systems

n/a	Involved in the study	
☐	☒	Antibodies
☐	☒	Eukaryotic cell lines
☒	☐	Palaeontology and archaeology
☐	☒	Animals and other organisms
☐	☒	Human research participants
☒	☐	Clinical data
☒	☐	Dual use research of concern

Methods

n/a	Involved in the study	
☒	☐	ChIP-seq
☒	☐	Flow cytometry
☒	☐	MRI-based neuroimaging

Antibodies

Antibodies used	nti-Mll1 (D6G8N) (Cell Signaling Technology Cat# 14197, RRID:AB_2688010)<br>anti-hSet1 (Bethyl Cat# A300-289A, RRID:AB_263413)<br>anti-H3K4me3 (Cell Signaling Technology Cat# 9727, RRID:AB_561095)<br>anti-H3K27me3 (Millipore Cat# 07-449, RRID:AB_310624)<br>anti-H3K4me2 (Millipore Cat# 07-030, RRID:AB_11213050)<br>anti-H3K4me1 (Millipore Cat# 07-436, RRID:AB_310614)<br>anti-H3 (Abcam Cat# ab1791, RRID:AB_302613)<br>anti-p21 (Santa Cruz Biotechnology Cat# sc-6246, RRID:AB_628073)<br>anti-cleaved Caspase-3 (Cell Signaling Technology Cat# 9661, RRID:AB_2341188)<br>anti-E-cadherin (BD Biosciences Cat# 610181, RRID:AB_397580)<br>anti-Mmp7 (Santa Cruz Biotechnology Cat# sc-26680, RRID:AB_2144469)<br>anti-GFP (Abcam Cat# ab6673, RRID:AB_305643)<br>anti-Ki67 (Thermo Fisher Scientific Cat# MA5-14520, RRID:AB_10979488)<br>anti-BrdU (Abcam Cat# ab6326, RRID:AB_305426)<br>anti-β-catenin (BD Biosciences Cat# 610153, RRID:AB_397554)<br>anti-TCF4 (Cell Signaling Technology Cat# 2569, RRID:AB_2199816)<br>anti-Lyz (Agilent Cat# A0099, RRID:AB_2341230)<br>anti-ITF (Santa Cruz Biotechnology Cat# sc-18272, RRID:AB_2287326)<br>anti-alpha tubulin (Sigma-Aldrich Cat# 00020911, RRID:AB_10013740)<br>anti-vinculin antibody (Sigma-Aldrich Cat# V9131, RRID:AB_477629)<br>rabbit monoclonal IgG isotype control (Cell Signaling Technology Cat# 3900, RRID:AB_1550038)<br>mouse monoclonal IgG isotype control (Santa Cruz Biotechnology Cat# sc-2025, RRID:AB_737182)<br>APC-conjugated anti 326 (EpCAM) antibody (eBioscience, Cat no. 17-5791-80)<br>Alexa-Fluor 700 CD45 antibody (BD, Cat no. 560693)<br>PE-conjugated anti-CD24 (eBioscience, Cat. no. 12-0242-81)<br>Alexa Fluor 488-donkey anti-goat IgG (Jackson ImmunoResearch Labs Cat# 705-545-147, RRID:AB_2336933)<br>Cy3 donkey anti-rabbit IgG (Jackson ImmunoResearch Labs Cat# 711-165-152, RRID:AB_2307443)<br>Cy5 donkey anti-mouse IgG (Jackson ImmunoResearch Labs Cat# 715-175-150, RRID:AB_2340819)<br>Dako EnVision+ System-HRP Labelled Polymer anti-rabbit (Agilent Cat# K4003, RRID: AB_2630375)<br>Dako EnVision+ System-HRP Labelled Polymer anti-mouse (Agilent Cat# K4001, RRID:AB_2827819)
Validation	All the antibodies used in this study were validated by the manufacturers for specific detection of the antigen and species reactivity and application. Specificity of histone modification antibodies was confirmed by dot blotting against synthetic peptides carrying the modifications of interest (Diagenode). H3K4me3/H3K27me3 antibodies were validated for ChIP by analyzing enrichment of positive and negative control regions (GAPDH TSS and Myoglobin Exon2, respectively); these controls are included in the manuscript (Supplementary Fig. 5a). The Mll1 antibody (D6G8N, validated by the manufacturer for Western Blotting, IP and immunofluorescence) was validated for ChIP by analyzing the Mll1 binding to positive and negative control regions (MECOM and TAL1+70, respectively); these controls are included in the manuscript (Fig. 5b, Supplementary Fig. 5c, d). Specificity of the Mll1 antibody in immunostaining was confirmed by the absence of staining in Mll1-deficient tissue (Fig. 2c, d, Supplementary Fig. 4a). Tcf4 and beta-catenin (BD) antibodies were validated for ChIP by including a negative control region (TAL1+70, Fig. 5c-d).

Eukaryotic cell lines

Policy information about cell lines

Cell line source(s)	Human colon cancer cell lines were purchased from ATCC:<br>DLD1 (CLS Cat# 300220/p23208_DLD-1, RRID:CVCL_0248)<br>Ls174T (CLS Cat# 300392/p720_LS-174T, RRID:CVCL_1384)<br>HEK293TN (RRID:CVCL_UL49), obtained from SBI System Biosciences
Authentication	Cell line identity was confirmed by Multiplex human Cell line Authentication (MCA, Multiplexion).
Mycoplasma contamination	Cell lines were not tested for mycoplasma contamination.
Commonly misidentified lines<br>(See ICLAC register)	No commonly misidentified cell lines were used in this study.

Animals and other organisms

Policy information about studies involving animals; ARRIVE guidelines recommended for reporting animal research

Laboratory animals	Mus musculus, Lgr5tm1(cre/ERT2)Cle, Ctnnb1tm1Mmt, Gt(ROSA)26Sortm1Sor, Kmt2atm1Afst, in C57BL/6N background, 4-6 weeks old at time of induction. Both females and males were analyzed.
Wild animals	The study did not involve wild animals.
Field-collected samples	The study did not involve field-collected samples.

Ethics oversight	All animal procedures were approved by the Landesamt für Gesundheit und Soziales (LaGeSo) Berlin with the number G101/18. Care and use of animals were performed according to the European and national regulations, published in the Official Journal of the European Union L 276/33, September 22, 2010.<br>Analysis of human colon cancer biopsies was approved by the ethic commission of the Friedrich-Alexander Universität Erlangen-Nürnberg (148_19 BC).

Note that full information on the approval of the study protocol must also be provided in the manuscript.

Human research participants

Policy information about studies involving human research participants

Population characteristics	Detailed patient characteristics (age and gender) are not known, as not relevant for the performed study. Biopsies derived from untreated patients (naive tumors).
Recruitment	8 naive patient-derived biopsies per tumor stage T0-T4 were obtained from M.Vieth, Pathology Bayreuth. No pre-selection criteria were applied.
Ethics oversight	Analysis of human naïve colon cancer biopsies was approved by the ethic commission of the Friedrich-Alexander Universität Erlangen-Nürnberg (148_19 BC). Informed consent was obtained from all patients.

Note that full information on the approval of the study protocol must also be provided in the manuscript.
